# Books (Are Not Like People): A Postdigital Fable

**DOI:** 10.1007/s42438-021-00256-2

**Published:** 2021-10-04

**Authors:** Eamon Costello, Tiziana Soverino, Prajakta Girme

**Affiliations:** 1 Dublin City University, Dublin, Ireland; 2 Dún Laoghaire Institute of Art, Design and Technology, Dublin, Ireland

**Keywords:** Speculative fiction, Socio-materialtiy, Educational futures, Distance education, Postdigital learning

## Abstract

What are books? In 2054, where reading and writing have been banned, a scholar in a dystopian academy known as University V might legitimately pose such a question. This article uses speculative fiction as a form of narrative enquiry to explore the socio-materiality of the iconic educational artefact of the textbook. It gives an empirical account of socio-material practices of textbook use (and non-use) gathered from a series of interviews with online distance education students. We analyse these interviews via speculative fiction. We engage in a sense-making activity of the student testimony by narrating their story, via a scholar looking back at our times from a post-literature future. We seek to contribute to a relative dearth of future studies that use real student data. We give an example of how speculative fiction may be used as a form of research method to analyse and interpret such data. In so doing, we seek to cast a light on current educational practices, to show how books and people are entangled. As people, objects and spaces of education intertwine, they call our attention to the interplay of form and function. They decentre the human actor. We attempt to show how form legitimates certain types of knowledge, certain people, indeed people themselves from other non-human actors. We conclude that knowledge is not disembodied, is not stable and is not locked up in books. In our final analysis, we conclude what may seem obviously true, that books are not like people.

## Introduction

The sound

Of a bell

Still reverberating

Or a blackbird

Calling

From the corner

Of a

Field

Asking you to wake

Into this life

Or inviting you

Deeper

Into the one

That waits (David Whyte, The Bell and the Blackbird, 2018)

**Fig. 1:**
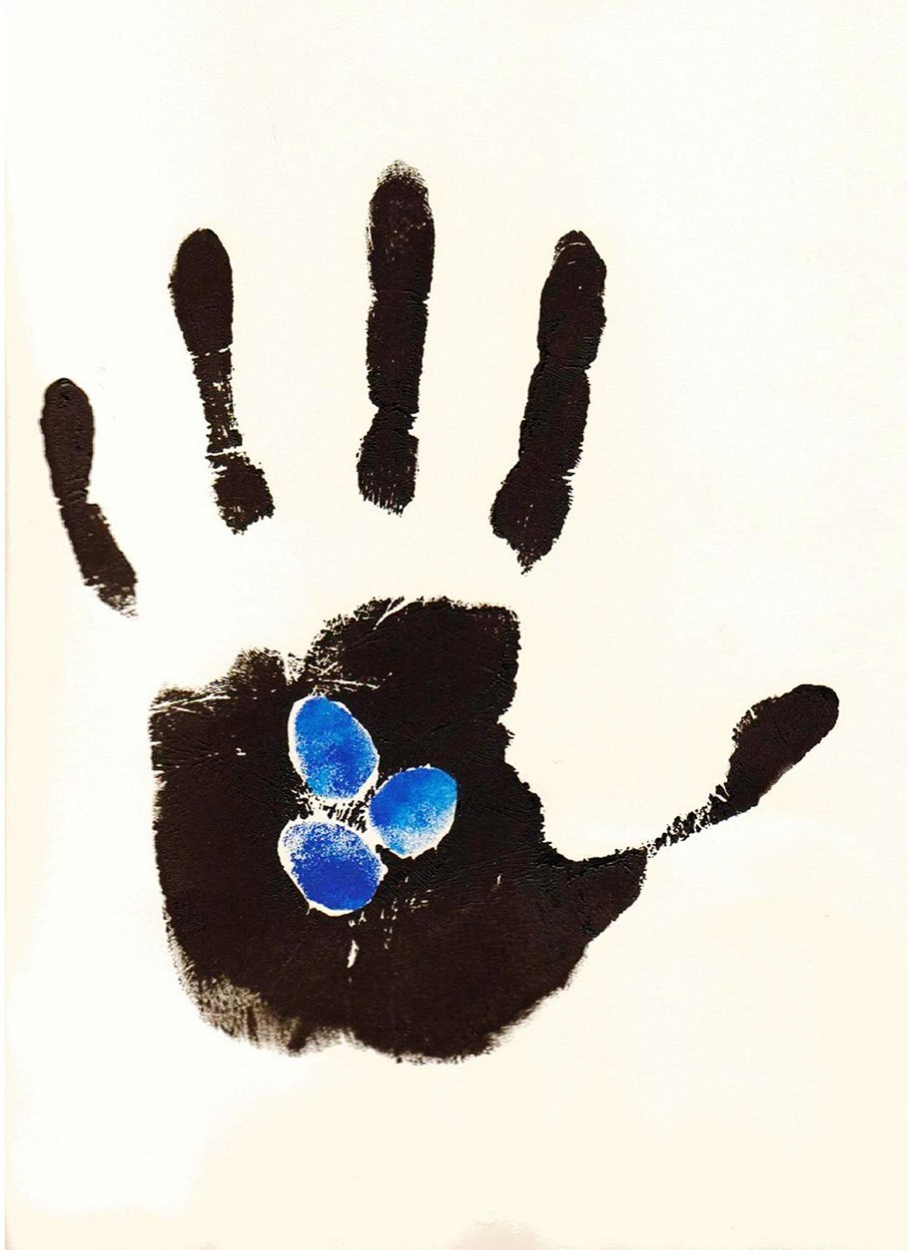
Her nest on me

Welcome to a story. Ideally, this piece would have no introduction (Spivak 1998). Stories generally do not introduce themselves. Stories try to plug the reader into the action right from the start. Academic forms, by contrast, have other conventions that should be followed such as signposting, introducing and summarizing the upcoming contents before going into detail about them, starting here in the ‘Introduction’ section. An academic text should not create suspense, or much worse, cause confusion, by concealing its ending, or indeed its purpose. A well-behaved and formed academic paper promises to explain, to clarify and to show. By implication, it claims not to hide, never to be coy and not to conceal anything in its pockets or in the depths of a shroud. As a crude generalisation of textual forms then, by placing them on a spectrum, we could say that one claims to hide; the other to show. This seems logical on the face of it. After all, fiction surely cannot show us more than facts? (Fig. 1).

There are no facts about the future, however. If facts exist, they are behind us. They are things we have agreed upon, proved and observed. They are the events, times, peoples and places that have been written on the page, chiselled in stone and beaten into metal. These histories have been written by their victors in such various forms. But who will write the future? And how, for there are no facts there yet? Particular representational tools are needed to peer into tomorrow (Veletsianos and Houlden 2020), ‘to consider what is probable, what is possible, and what is preferable’ (Kupferman 2020). They may be tools that transgress, that queer and that trouble text (Bayne et al. 2020; Phillips and Kara 2021). Transgressive perhaps because ‘in the age of validity, inter-rater reliability and evidence-based research, it can seem subversive when researchers “tell stories” (rather than “write reports”, “produce findings” or “demonstrate effectiveness”)’ (Macgilchrist 2020).

Stories allow us freedom of ideas and we need good ideas to have ideas with (Haraway 1991a). Such ideas might be ‘the friction of tyres on roads or the noisy hum of daily life’ (Macgilchrist 2021). When Lucinda Williams (2008) sings ‘car wheels on a gravel road’, we know what she means. She means the crunch of a vehicle turning on a spilled stone surface, of someone coming, of dust rising in the train of movement. Hopefully, if you listen carefully enough to this article, you will also hear something approaching and in particular you should listen out for a bell.

We use speculative fiction (Ross 2017) here, as a type of narrative enquiry (Clandinin and Rosiek 2019), drawn from a posthuman methodological scabbard, most appropriate for cleaving embodied and disembodied postdigital pedagogies (Bayne 2018; Lakind and Adsit-Morris 2018; Knox 2019) and for peering into the classrooms of the future (Selwyn et al. 2020; Macgilchrist et al. 2020; Costello et al. 2020; Costello and Girme 2021; Cox 2021). Selwyn et al. (2020) give a useful account of their approach to writing speculative fiction as research. Explications of methodological workings function to legitimate research as replicable, or in qualitative terms, ‘trustworthy’. Although stories are not true or trustworthy, and their approaches are not always designed to be replicable, we will nonetheless return to this issue in the narrative itself via fragments of the methodology section of a manuscript known as the lost codex (see the section ‘The Codex’ below).

One thematic function of our tale will be to find the University of the Future. It may be in Border U, a university built around boundaries and migration (Gildersleeve and Sifuentez 2021). It may be the University of We—a place not composed of regulations, not of administrative law or codes of conduct but as manifestation, or outward essence, of ‘the implicit or deeper layers of character, care, and the demand from the other’ (Nørgård et al. 2020). The future may be the Spectral University Su (Cox and Levine 2016) or the Academetron composed of ‘uncanny spaces, in which new, anxiety-provoking yet rich understandings of the nature of being in a digital age can be confronted’ (Bayne 2008). The future may be in the ‘ecological university’ which is a ‘university neither in-itself (the research university) nor for-itself (the entrepreneurial university) but for-others’ (Barnett 2011, 2021). Who, or even what, ‘others’ are is something we will need to figure out, for in an ecological university separateness is suspect. The ecological university is a place where ‘we are not separate from nature, we are nature, we are in the world not outside it’ (Facer 2021). All of tomorrow’s universities need imagining, but here we use speculative fiction to venture deeper and further into imagination and into the future (perhaps dangerously deep and far). You just need to listen out for a bell. It is calling you, as the poet David Whyte (2018: 23) tells us, ‘to wake into this life’ or is ‘inviting you deeper into the one that waits’.

In the fiction that follows, we divide our time between historical events. We visit the world’s first copyright case, an argument between two monks over the copying of a manuscript in sixth century Ireland (Ó Baoill 2018), one of the first recorded disputes over intellectual property (O’ Donovan 2014). It heralded the dawn of books as material embodiments of ideas that could be fought over. Following this early manifestation of the written word, we travel to University V in the year 2054, a world where writing has been outlawed (Costello et al. 2020; Costello and Girme, 2021). A scholar in this dystopian academy has stumbled upon student testimonies from a research study. Our protagonist attempts to piece together fragments of this strange codex to gain insight into the world we live in now.

We speculate, from this future, upon the nature of knowledge, with which the core identity of universities is closely entangled (de la Bellacasa 2012). We take books as an archetypal knowledge artefact—something that represents, both symbolically and practically, what we know and how we know it. Our research question then is concerned with knowledge of the present as seen from the future through the accoutrements and processes of knowledge production. Hence, it asks: What are books? More specifically, but perhaps obliquely, we ask: Are books like people? Text, of which textbooks are comprised and represent, has been troubled (Bayne et al. 2020; Derrida 1998) and here we attempt to unpack this claim. We do so through careful and attentive depictions of students. Such students would not be datafied but perhaps storified instead (MacLure 2013). The speculative fiction that follows hence attempts to contribute a socio-material account of students’ lived reality of textbook use (and non-use).

## The Bell

**Fig. 2:**
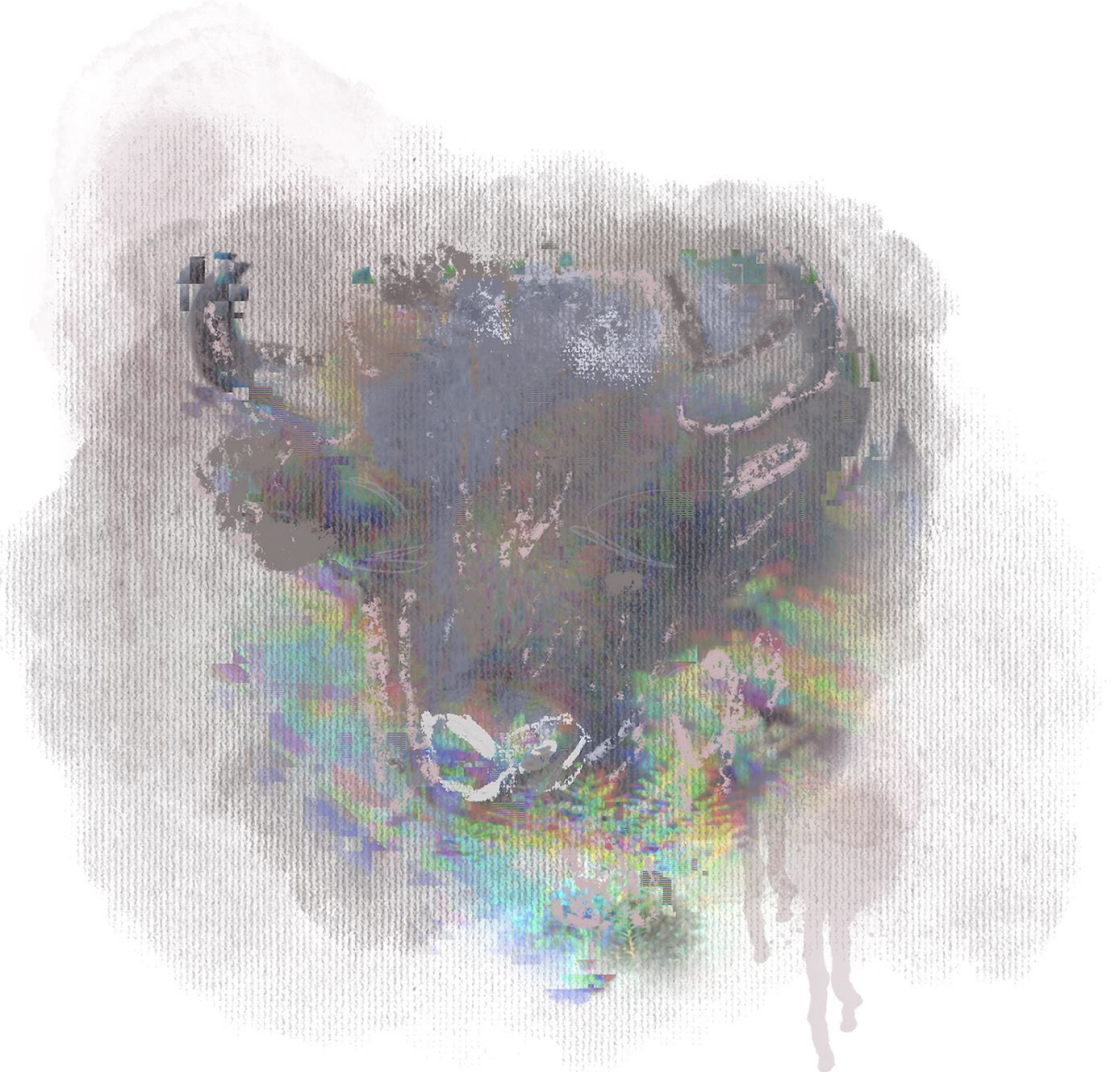
Cattle pasture

I was late. I thought I had been taking a shortcut, but no. I was so close though. Close enough to see the spires of my destination and to hear its bells crashing in my ears. But I was not there yet and I had ground to cover and choices to make. Closest to me was the cattle pasture (Fig. 2). The tips of the horns of the cows curved up from the meadow, their rusty heads dipping below the grass line. I could cut through there—they seemed harmless.

A shorter route was through the flax field. There were no large horned animals in it, just a sea of flowers, purple petals held upon yellow discs, atop stems bent this way and that. These were the plants that grew the flax, for the linen of the linothorax, of the Archangels of University V (Aldrete et al. 2013).

Flax for linothorax linen must be unadulterated: ‘for she was given fine linen to wear, bright and pure’ (Revelation 19: 8). No foreign chemicals can enter into the flax’s autotrophic cycle and nothing can transgress the boundaries of its enclosure, no interlopers, no contaminants. Only Archangels themselves can walk in that field.

The bell summoned me again from the towers of the campus, calling me to my academic work. High Professors were assembling and I could not keep them waiting. This semester’s Life-Credit-Average was at stake and the datafications we painted in this year were particularly vivid and colourful. If I went the long way around I would be late, but danger awaited if I cut through the fields. So what was it to be? The wrath of a High Professor in her theatre? Or the breath of the cloak of a University Archangel billowing down upon me?

The bell again, feeding me further into the panic. ‘Imagination will find a way. Imagination will find a way’, I told myself, squeezing my eyes shut but I could feel my throat constricting. ‘Imagination-will-find-a-way. Imagination-will-find-a-way. Imagination-will-find-a-way.’ The bell was ringing again and its chime cut into me and began to buzz and shimmer in a swell of bronze striations. It did not sound like an instrument of clarion, but the voice of a creature, its beak submerged in the metal. I followed the sound of the bell until it should have ended but some part of it seemed to still resonate. And then, I could hear something else—a blackbird.

There it was, pausing in its song, to turn its head and eye me, pausing in a gap I had not noticed before. There was a small space between the fields, a narrow path that must have been used by the University farm labourers. I bolted down this opening, my heart pounding, scattering the bird before me. ‘Thank you blackbird’, I cried as she flew up, ‘Thank you so much’.

## Scriptorium

I reached the grand theatre just in time and made my way down the staircase to the lower level of the middle tier of the scriptorium. I moved quietly to my place. Arranging my things across the desk at the front of my platform, I fitted myself into the harness and plugged myself in.

High Professor A was walking around the room. She had stepped down from her stage and was weaving her way between the students, through all the spaces that were left between us. Her red hair unsettled about her, in curls over her speckled shoulders. I thought some light seemed to follow her, or she it.

I felt High Professor A’s presence calming me, my work absorbing me, my heart rate sinking. Why was she called Professor A? There were rumours. It was said that she hacked her own name. That she cleaved off the other letters in an extreme act, forging herself a scholastinym that would move her further up the alphabetised author list of an academic paper. There were other stories. A student, from one of the lower plateaus of one graduate caste, claimed she actually had a longer name and would reveal more letters of herself to students as they progressed through the plateaus.

Hacking her own name, brutal as it sounded, was still the more plausible explanation. High Professors were well known for their physical augmentations, the mods that allowed them to see and hear us in such high fidelity, in ever greater pixel counts (Costello and Girme 2021). Those were standard human practices from the dawn of time: to sculpt flesh, pierce, paint and augment the body. But to mutilate one’s own name? Only one of the most brilliant minds of its time, in the greatest university on earth, could conceive of, and execute such a thing. Thus, via Ockham’s razor, was the truth of it.

## The Codex

I put my head down and got back to work. I took up my tools carefully, observing the safety protocols that were almost as good as habits now. After ensuring I was fully in harness, I authenticated myself by resting my tongue on the sensor. Once I had entered this first gate I entered my numbers, my symbols, my icons and then my birth date as it had been numerated upon the University V academic calendar. Lastly, I entered my pedagogy, that which I had been anointed with the day High Professor A stroked the chrism down my forehead with her thumb. It was the name I would have until I walked through the fires of graduation. The final gate parted and I accessed the Lost Codex, slowly bringing up one of its pages behind the protective gauze. I allowed the deepmind hive AI to mediate its words to me, so that I did not have to look upon them directly.

The Lost Codex survived only in fragments. It appeared to comprise some set of academic rituals that had been abandoned before they had been fully enacted (Costello et al. 2020). The sections were broken and it was incredibly complex if not impossible to ever piece them together. It was unlikely to ever be properly decoded and hence High Professors gave it as an exercise for students at my level. There was little risk involved for us in working on it. Some commentators dated it to just two years prior to the first pandemic, finding markers of a time when humanity was still unknown to itself, when it was mired deeply in its own dark reflections, when knowledge was on the cusp of collapse, when the text had become troubled (Bayne et al. 2020) and just before writing had ended forever (Costello et al. 2020).

I was not so sure, however. It seemed to me that there were clues that this might have come from a brighter period than had been described by historians. I saw glimpses of other worlds in the codex, ones that could not have belonged to such a dark era.

It was just so impenetrable though. I shuffled some of the fragments on the screen: ‘Limitations of the study’, ‘thematic analysis’, ‘Braun and Clarke (2006)’, ‘creative commons’, ‘accessibility’, ‘distance education’, ‘focus groups’, ‘(Authors Forthcoming)’, ‘qualitative methods’, ‘(*n* = 8)’, ‘OER-enabled pedagogy (Bali et al. 2020; Clinton-Lisell et al. 2021)’. None of this made any sense. Parts of the rituals seemed to revolve around some obsession with ownership, or stewardship, of large sheaves of words known as ‘textbooks’. Some people appeared to have a kinship with these entities, while yet others deemed them objects of enmity. They could be reviled or revered in equal measure it seemed.

The words would stay in the pages, always on the lines, but then, through reading, the adherents could imbibe or construe them into themselves somehow. It was not my job to look too deeply into the occultism of reading, however. The deepmind hive AI was here with me, protecting me from this. I just needed to demonstrate my mastery of the tools I had been learning. I just needed to scratch but one fingernail of paint from the mysteries of the codex.

What were textbooks? Where did they live? Did they replicate themselves mindlessly as viruses, or as animals do through love? And in what hierarchies did they exist? These were surely the types of questions that could unlock any mystery. I decided to go and find the source of the trouble. I needed to go ‘back in time’ (Welch 2001). There must be a place in time, I reasoned, where hardbacks and thick spines were first fitted to books. But also there must have been another place in time when these things became something more, when their contents jumped down from between the lines of the pages to write themselves upon us. I would go there.

## The Work

I found myself in a ribbon valley. I saw a round tower and a window. Something moved inside. I thought about what High Professor A would say: ‘You must never ask anyone for a single word. Just let people tell you. Listen and they will speak unbiddenly. Sit them in an unbearable silence. If you ask, they will give. Just wait. Give it time and then, when they open their mouths, you can reach in and steal them.’

I could not understand what she was saying much of the time. I just heard the deep conviction in her voice. In this case, I knew she seemed to ask me to wait and so I did. Sure enough, after a while, I heard something: a monk shuffling and then kneeling inside the tower. I felt myself putting his cowl over my head. I felt my knees on a cold floor. I heard him murmur and his story started to become mine.

In my stone cell, I sat and prayed. And every hour I took a break. I stood up and tried to activate as many muscles as possible, without fixation, and always with the aim of resettling myself back into the contemplative state as quickly as possible. During one such interlude, I would stretch my left arm out the window, and then, in the next hour’s break, the right. Such were the confines of my stone room in the tower that I had not the luxury of any more movement. Nor did I need any. I had enough space to give my body the shakes and stretches it needed to stave off any sores, pains, or the pull of sleep.

I stood, shook my robe, shuffled my feet, moved my head from side to side and pushed my arm out the window, circling my hand at the wrist. Turning my hand up towards the sky I felt something gently grasp my finger. It was a blackbird. As she perched there in some uncertainty, I tried to explain her misconception. ‘This tower is not a tree but an assemblage of stone’, I said, ‘I am not a knotted pattern on its bark but a man in a hollow inside the stone. That is not a branch but an arm of my body. That is not a twig but a finger of my hand.’

The blackbird moved its head to one side, keeping its eye on me.

‘You can stay a little while’, I said, ‘You can rest for a bit until I resettle and ready my body again for the work.’

The blackbird heard my voice and moved towards me. She settled onto my palm, her body warm and soft in its hollow. I waited and she closed her eyes and became still. The sun left and the frostbit stars into the skin of a long night that I waited for her. In the morning, there were five blue eggs in my hand, and she flew off to find twigs, grass and moss to weave a nest. When she was gone I kept my arm straight but I curled my fingers over the palm to shield the eggs from the wind and the gaze of buzzards and crows. When the nest was built, she sat and I waited for the eggs to hatch. As I did, she brought me blackberries and hazelnuts and I sipped water from her beak. Once the eggs hatched she was too busy for me and fed only her chicks. I waited for them to fledge, and then, one day, she was gone. All that was left was her nest on me (Fig. 1). I waited for the wind to blow it away. Its bits and pieces it left. When it was gone I took my hand back into the cell and I resumed my prayer.

I felt the stillness of his cell and realised that it was all the monk could tell me. It was a dead end. There was nothing here relating to textbooks, to students, nothing that could help unpick the codex. I was failing in my task, falling behind in my work. After all that effort and patience and energy, I was no further along.

I saw the blackbird looking at me and I tried to reason with her: ‘I am not the monk. The story is over. The tale is told. I am a student. I am from another time.’ She alighted on my shoulder and I let her wait there a while. ‘Okay’, I said, ‘you can stay there a while but *I* have work to do and it cannot wait’.

## To Each Cow its Calf

I found myself on a hilltop, wind sweeping waves of grass about me. The bird sought shelter in the crook of my neck. ‘Come on then’, I said, ‘Come under my cowl.’ It was then I noticed before me another monk and with him a warlord. The monk gestured towards me and spoke rapidly in a strange babble.

What language was this? Why was it construed so oddly? Why had they no word for ‘yes’ or ‘no’? Had one and zero not been made yet? Had the digital not even been pre-figured? Was no-one talking of Bayes yet? (Birhane 2021) I was very far back in time, perhaps dangerously deep. William of Ockham had not yet scribed his laws of logic. He had not yet created the binaries that we could use to give ourselves machines, sexualities and all other manners of things to live in. We had not yet his axia. All his nominalism was still dreaming, and wherever I looked now I saw this unconfiguration in such vastness that it could ‘take days to describe’ (Watts 2013: 21).

This language did not have words for ownership. Its etchings were as crude as scratches upon the rock. Instead of ‘to have’ it merely croaked ‘to be at’:

Tá leabhair agam;

There is a book at me. (Ó Baoill 2018)

I had, however, now quickly learned enough of this *teanga*, this tongue, that I could understand what the other monk was saying as he pleaded with the warlord in a frantic tone.

‘Give me judgement, give me justice my lord. If you be the strong man, if divine law can preserve what I have written, tell us: Who is right here?’

He pointed at me: ‘This leary-eyed monk stole my work, copying my words in his cell at night.’

‘I thought they were the words of God’, I replied, ‘I only wish to serve Him, to preserve the words of His will. I simply wanted to copy the scriptures so that I, and everyone, may better know what He wants us to do.’

‘Lies!’ screamed the monk, ‘You lie! Thief, thief, thief! You steal my work for your own glory, for yourself. You slip the facsimile under your garments. I see it through your apparel as a mark burnt upon your chest. Shame! Shame!’

The warlord, who had been listening quietly, stepped between us. He raised his hand gently, but his whole arm seemed to move, from a twist of his torso, that called upon muscle all up his back to his shoulders. The other monk and I fell silent, and let the High King of Ireland Diarmait Mac Cerbhaill, deliver his ruling:

To each cow its calf;

To each book its copy. (Ó Baoill 2018)

I opened my robe and took out the manuscript I had spent so many nights faithfully transcribing. I handed it over. It was done. This short trial was over but I sensed war was coming. The battle for this book was but a skirmish (Weller 2014). I had learned enough, and I felt myself ascending. I left my body behind and the figures of the three men circled smaller and smaller below me.

I heard them pronounce exile upon me as I rose. As if that would help! The cat was well and truly out of the bag. There is no punishment for plagiarism, I wanted to tell them, this blackbird could steal your voice as soon as you speak if she wanted to. Cup your hands to your mouth and hear the mimicry in your own echo. Tell the dew to give you back your reflection.

I had now experienced how books came to be (O’Donovan 2014). These assemblages, of liquid colour on sheets of animal skin, were vessels of a vital message, perhaps even the most important message of all. But much more than all of that, books were no longer selfless. They had an identity now, through the doppelgangers of their transcriptions. Through these uncanny ghosts (Bayne 2008; Cox and Levine 2016), they had been given unsettled and hence true selves. I was lifted by high hopes that this would help me in my work and so I hastened back to the codex.

## Horrible Sociology Book

I brought up a fragment carefully:

Interviewer 2: I can’t remember, maybe it was… it was [Participant 2] mentioned about getting books from other students. And, and how did you… how did that come about? How did you network with other students…?

Participant 2: It was the buying and selling forum on Loop on the Humanities Hub page, and she put them up towards the end of the year and I said, I know I’m gonna need those, since I had checked the booklist from the previous year. So I edged my bet [sic] but even if I needed four… I think I bought ten off her in total, and I reckoned even if I had bought four or five, I’d still be saving a lot of money going by what she was selling for, and then it happened that her husband lived nearby and we met up at the petrol station and he literally just handed me my pile of books and I handed him the money. See you later, and that was that.

Strange shapes dripped from her testimony like cooling wax. There was something exciting about that meeting for her: ‘see you later, and that was that’. Had she wanted something more from this man?

**Fig. 3:**
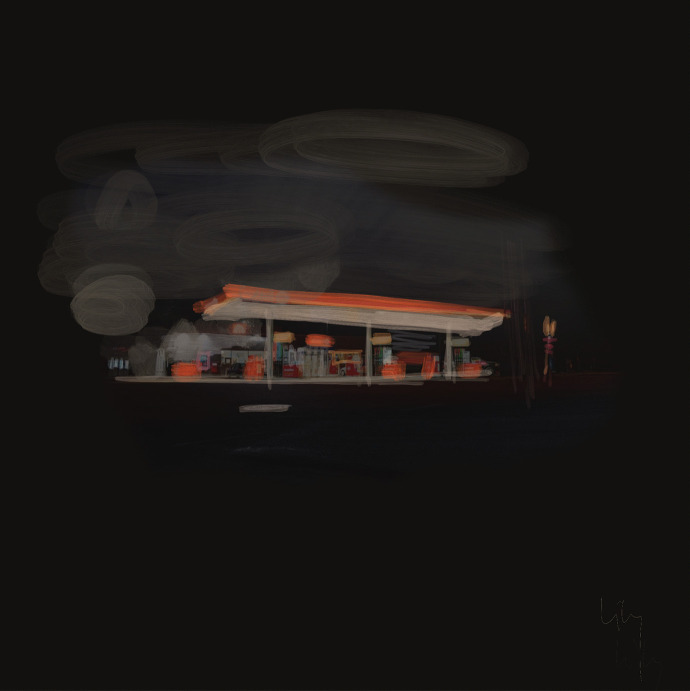
A waypoint

What was a ‘petrol station’? All I had determined so far was that some kind of liquid was sold there, a substance carefully called up by squeezing triggers of silver guns on long black tubes. Maybe a petrol station was a waypoint (Fig. 3), a watering hole, a safe space and petrol a drink proffered there to revive the weary. It might be something they sipped, while exchanging books, safe and surrounded by tarmac. Petrol sounded both natural and synthetic. Was it a nectar? Was it harvested by a host of tiny flying machines, borne on silver insect wings; sticky pollen free-riding, flower to flower, on glass and carbon cilia running down their legs? Did students sit outside at this station, on asphalt, oozing in the sun, amongst the piles of books, the smell of chamomile and all the flowers of the field drifting up from paper cups that brimmed with clear sparkling petrol?

I do. I do think they’re…for the modules that I have done, I found them to be invaluable. I’m just thinking, the beginning’s one, like I cannot wait to be rid of it. It is massive, you’d knock someone out with it, but it was always a starting point it’s like any assignment that we have to do it was the best place to go to, because it would have a massive amount of information about that topic, and it would give you a really good overview before you start to narrow in on where you’re gonna go.

‘It’s massive, you’d knock someone out with it.’ How curious. Did they mean physically or was there more to this? Were books weapons? If you just hit someone with the right words? Or was it more nuanced? Were they teaching students to use books to put various emotions into each other?

I suppose the difference with Philosophy is, it’s, it’s not, it’s not something that you just read once for an essay or something you… you know, they’re just books you might dip into and read just because you’re reading, you know, just for pleasure as well.

I saw the student ‘dip into’ the book. This was something I could not do. I could never feel a book against my skin like this. The deepmind hive AI mediated their words to me, as slow drips of a tincture. It kept me safe. But for some reason, even though I did feel so happy, there was some tinge of something else. Maybe it was my sympathy for this student, way back in that time and all of its conditions.

For Psychology, except for the kind of the… classic theories, it’s pretty much all journal articles, so it’s everything that’s available through the [University] library and the databases that we have [inaudible], which is really frustrating then when you come across an article, and the title is exactly what I’ve been looking for and you click into it and we don’t have access because we rotate on [inaudible] [University], and [University] doesn’t have visitor access, and it breaks your heart a little bit….It just happens to be exactly what you are looking for…

This seemed clearer. It was about the inaccessibility of words and their assemblages, about their uncanny nature. It seemed that some forms of writing were already being locked up, concealed behind opaque walls, abstracted from us: click but don’t touch. The dangers of written words were perhaps already revealing themselves by this time in history.

There was more in this fragment that I could not fathom, however. Why did she say ‘exactly what *I’ve* been looking for’ and then switch to ‘exactly what *you* are looking for’? Did she know what *I* was looking for? Could she see *me*? See me not as I felt myself to be, feeling and fumbling for things, but as someone who knew ‘exactly’ what I needed? It ‘breaks your heart a little bit’, she had said. How could it break it but a little? How would not being able to get exactly what you were looking for not pull your whole heart asunder?

Eh, I have an educational psychology one [book] that I had an elaborate kind of way of getting to actually get it to me, ‘cause it was quite expensive and I found it for sale in Italy and I had it bought for me in Italy and then they mailed it to me so and it’s, it’ s quite useful one and I might go on and specialize in that, for that’s moved up to the top shelf, it is more value book, now the rest are all still on the second shelf.

This book had come to the person via some ‘elaborate’ supply chain of events, people and places. It was ‘useful’ for she could somehow ‘specialise’ herself in it. As it specialised her, it had ‘moved up’ to the top shelf, sorting itself amongst other books, rising through the emotional hierarchy. Just as we are raised, here in University V, through the plateaus and the castes, semester by semester, in the changing seasons of the academic calendar that ever cycles us upon the meticulously seeded glitch.

It’s hard to get rid of those books afterwards. I would finish with that horrible sociology book last year and I thought it would be easy enough to offload it, because we all had to get it, but people could get it like for apparently, a really cheap now [inaudible] book whatever depository, whatever or something, so nobody was interested. So, it’s sitting on the floor here.

This fragment must join with the petrol station one. Here again adherents were ‘sitting’ amongst the books. This must be why books had such odd physicality, such stubborn form in the face of the digital. There was no need for these lumps of paper, ink, cardboard and glue unless it was precisely their blockiness that was desirable. These things must have had weight as well as mass, perhaps even scents. They may have had tongues and grooves on their alternate surfaces that could slot into each other, so that they could be built into walls and then further fashioned into larger structures. Stacked and connected towers of books could become windbreakers to set upon the ground or armchairs one could sink into.

**Fig. 4:**
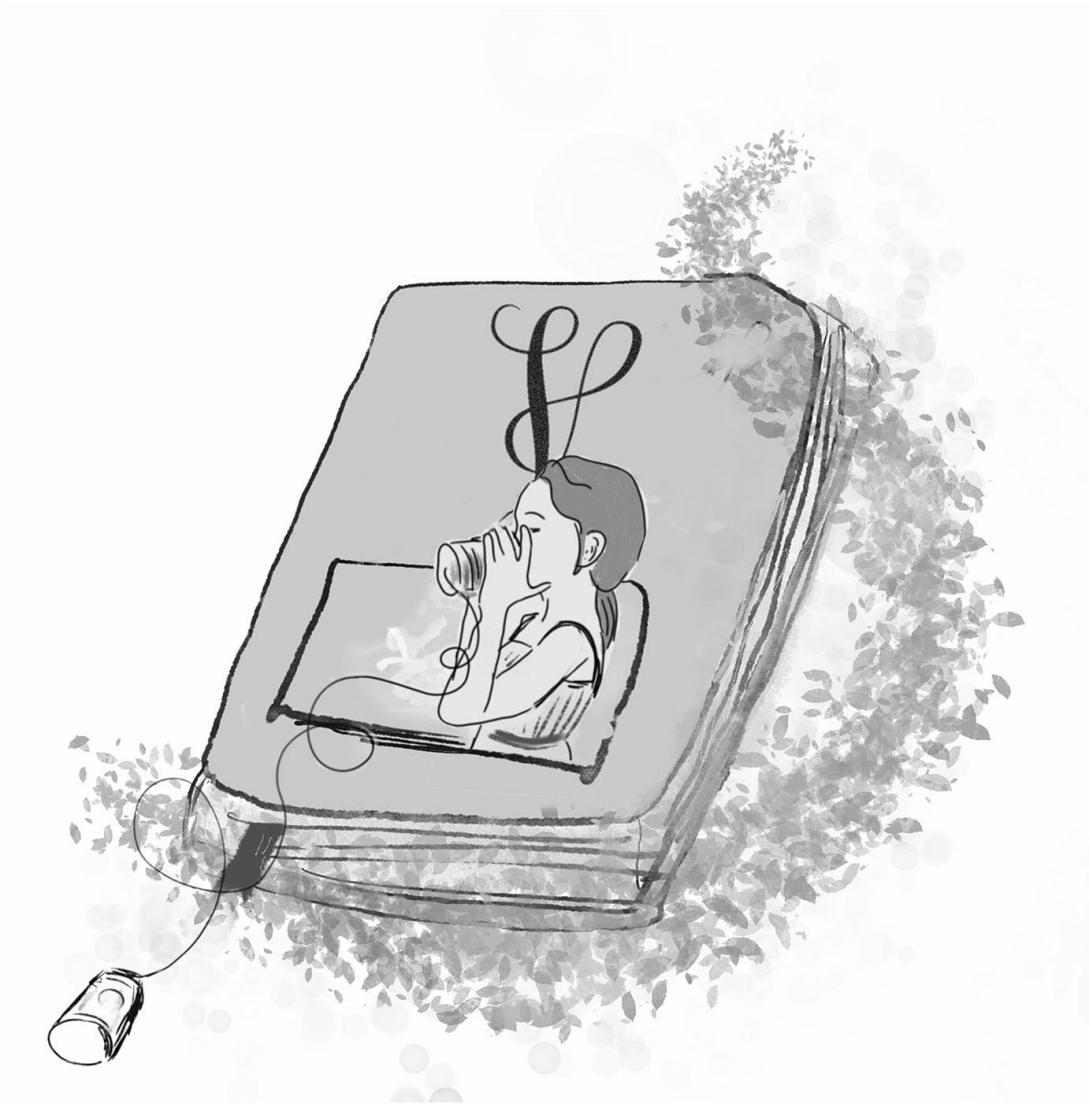
‘Horrible sociology book’

I pondered the ‘horrible sociology book’ (Fig. 4) that ‘nobody was interested in’ because of its ‘really cheap’ copies. Perhaps this was how reading was abandoned, just as this book, ‘sitting on the floor’ with nothing but a dream of fingers on its spine.

Participant 2: Give us them [the reading lists] in advance of the year. Straight up, give me a nice list, stick it somewhere….

Participant 3: Yeah!

Participant 2: So I can go on a hunt for the whole summer…, And I find it really frustrating, because just when I have to pay out all my module fees in September and then, bang, I’m getting a book list that, if I buy it new, you’re talking three to four hundred quid. I’m really, really frustrated!

I had no idea what any of this meant so I skipped over it. It seemed complete gibberish. Something about hunting and students shouting. Books as some form of expensive trophies? I was getting tired now of all the analysis and I felt fuzzy-headed. The codex could do this to you. I tried to soldier on for a little longer.

… so I actually don’t use textbooks very often…I buy them because I, I suppose I’m afraid of missing out, but I tend not to use them in the end…

They had fears that they would miss something from books even if they did not use them. Or perhaps they did not want to allow themselves to be used by them. There was some longing they had to be near the words even when they could not bear to look upon them.

Sometimes I’d wait and see if I think I’m going to use this, especially in my first year, I went to the library and if the book had a teeny-weeny print that I know I’m not gonna be able to read anyway I’m not gonna buy it because I know I’m not gonna ever look at it you know…

In this fragment, the adherent seemed to get close to the book, via the library, but then be repulsed by some visceral aspect of it, and withdraw in disgust. There must have been some painful element to access something in small physical form. It became too sharp for her as it diminished.

I had so many ideas now that I almost felt I was them. I felt a chaotic electrically infused tide of blood rushing around me. I tried to wade out of its sticky and dense substance. I needed to find my way back. I need to come back to myself.

## Blackbird

I unplugged myself and left the scriptorium. It was getting dark as I walked back across the campus. I heard a song come into my head by Lackland John (2021): ‘Books are not like people’, he sang strangely. I passed an oak tree and saw pink blotches on its bark. ‘What is painting you in its colours?’ I said. I looked up, and in the distance, I saw the lights of a skyscraper that were causing the effect. Its neon windows were winking as people left its rooms for the night. I thought about what might be in there, about who those people might be. I fancied that they might be librarians in ancient times, changing shifts, moving books between floors. I thought that there must be other trees out there, their barks being slowly painted in pink rectangles, as the sun faded and the neon city started to blink its eyes open.

I had studied all day and yet I felt I had learned so little. The codex seemed to just set me upon a sea of myself sometimes. And I was not quite back to myself yet. There were some binds that had yet to loosen that still wrapped me in the past. I was close but I felt I was still a day away.

I pulled my cowl up over my head. Why was that there? Everything seemed to be melting. I heard a bell. I saw someone running, struggling. I felt their pain as if it were in my hand, as if they sat on my palm and I closed my fingers over the top of it, to protect it from harms, from rooks, from raptors, from the elements and from turning in on itself.

I thought I saw an encoding of a younger me, in another life. It was a different person but it was still me. The person was full of panic and indecision.

‘Don’t worry young me’, I said. ‘You have time on your side. Everything will work itself out. You are right. Imagination *will* find a way. It will find you. Here - in the meantime, you can have this.’

**Fig. 5:**
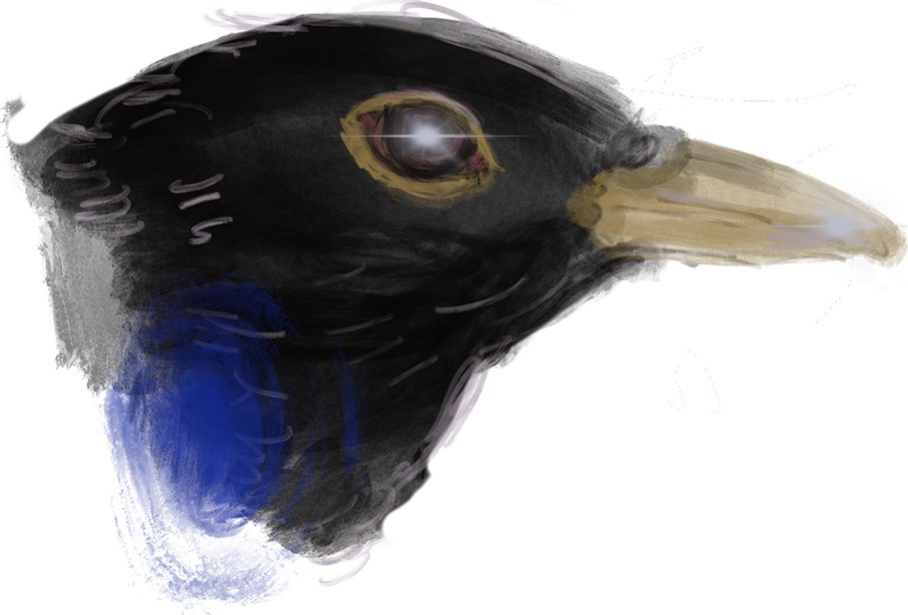
This is not the end

I took the blackbird down from my shoulder and I set her down by a corner, where two fields almost met, but were bisected by a narrow path. ‘Goodbye’, I said to them both, ‘but don’t worry, this is not the end’ (Fig. 5).

## Discussion

A contention of the postdigital is that ‘contemporary student practices with technology are complex entanglements between physical and digital technologies, spaces, activities, and time’ (Jandrić et al. 2018: 896). In this piece, we contribute to postdigital theories of education, by substantiating such claims, via testimonies of students’ lived use of textbooks. We tangle our participants as deeply as we can within these objects. Instead of trying to cleave them from their educational accoutrements, we weave them further in. We avoided, however, the books spilling too far out, as full posthuman characters in their own right, unsanitised and unsafe, because, as the books took on postdigital form, they hinted at tales of desire, being and embodiment. The testimony of the students became at times confused with the testimony of the books, so we should be clear about one thing: books are not like people. A book cannot tell its own tale, cannot speak of that which is bound within its covers. It would not ‘insist on telling stories of desire, of complexity, of variegation, of promising myself one thing at night, and doing another in the morning’ (Tuck and Ree 2013: 648).

In this speculative fiction, we encountered a manuscript made from the skin of a cow: the first book. A worded object trying to become flesh again. Trying to make itself desirable, an embodiment of God, of ideas and of hope, enough of these that men would fight over it. Or at least that is how they wrote the story, retold here, of the first copyright dispute. This incident is one of the first recorded incidents of an intellectual property dispute (O’Donovan 2014). It was settled in favour of the original owner of the book, against the arguments of the copyist. This is an enduring dispute. We could take the Sci-Hub platform as a contemporary analogy. It provides free access to academic and scientific publications that are proprietary in nature and usually cost money to access. It is illegal in many jurisdictions although it has been argued in courts in countries such as India that scientists are reliant upon it (Elbakyan and Bozkurt 2021). During the pandemic, it is reported to have a huge surge in access to its articles particularly to research on COVID-19 (Elbakyan and Bozkurt 2021). The ‘battle for open’, however, as Weller (2014) puts it, can sometimes be beset by a sense of pyrrhic victory. To pose this postdigitally, perhaps unfettered digital access to effusions of content itself contributes to a ‘fantasy of a weightless and untethered digital education’ (Bayne et al. 2020: 61).

The unseemly copyright dispute pre-figured a world of white male ‘book knowledge’. Such knowledge may be pitted against the basic wisdom of some communities that is needed to survive (Collins 1989). Such survival wisdom may be generated in the face of tenacious epistemologies propagated by those ‘who readily sacrifice their intelligence to their prejudices and possess more knowledge than wisdom’ (Collins 1989). As this translates to the continued assembly of the University and its knowledge bases, animals (non-human or otherwise) may gather to bite that hand that feeds them by challenging ‘the latent theory of change that research, more academic knowledge, will somehow innately contribute to the improvement of tribes, communities, youth, schools, etc.’ (Tuck 2018).

A famous cybernetic engineer once developed cyborg creatures which she lovingly dubbed as her epistemic blasphemies (Haraway 1991b). People can be allowed some egoic leeway when it comes to their children of course. We are only human. Some blasphemic characters might even have narrated themselves into this article, as strange agents that are not of the types of knowledge they are supposed to be.

We teach to transgress (hooks 2014), just as we think to unlearn, for ‘in the beginner’s mind there are many possibilities but, in the expert’s, there are few’ (Suzuki 1970). Indigenous knowledge knows it. Book lovers, slow under the weight of words, are even catching on, glimpsing the idea of agency as a characteristic of both human and non-human phenomena. Indeed, most strangely of all, it may be ‘that stories are themselves agents with which we need to consider our ethical relations’ (Rosiek and Snyder 2020). Although at the outset we tried to invoke the University of the Future, commanding the story to take us there, all we found was a scholar daydreaming of us trapped back here in the present—and what a lovely dream it was.

In the twilight of an academic article, sections emerge such as acknowledgements, author contributions, ethics statements or even, very late in the day, citation diversity statements (Zurn et al. 2020; Dworkin et al. 2020). Stories tend not to have these. They do not call attention to preponderances of particular sets of people whose ideas and types of knowledge they legitimate through their citation practices. Stories hide things from you. They fabulate. They cannot cite, create might, rhyme or reason. When a story cites a song, just as it is being sung, what does that say? The voice has not been captured and peer reviewed yet. Her testimony has never been validated and the forms of such knowledge are not the right ones. We cannot trust her as it all happens too immediately and too closely:

Darling remember, when you come to me

I’m the pretender; I’m not what I’m supposed to be

But who could know if I’m a traitor?

Time’s the revelator (Welch 2001)

If we had the time, we could get to a character diversity section. Professor A would seem to be an able-bodied woman. As to her race see the previous chapter (Costello and Girme 2021) where she becomes embroiled in the University student until her gender and her body becomes his body and his skin. As the reader is beguiled into thinking she is a woman in this tale just told, thus the story genders its protagonist, the affable scholar. Although he has no name or pronouns up to this point his male gaze may fix his teacher as a sexual object, a crush of beauty intellectual and physical (Mulvey 1975). Professor A is tangled up in these by him, by herself and the rumours of the web she sits in. The lesser characters are easier: some men shouting; a female bird who guards her eggs and makes decisions about who to tend to; the long-horned creatures in the pastures may sit, languidly, in the ancient Egyptian set of animals that are ‘seen as good mothers, including the lioness, cow, and hippopotamus’ (Gucciardi 2013). And as to the gender of the bell, University V and perhaps most interestingly of all, the neon city, well that is a story for another day.

We did not consider social class here, as pointed out by Reviewer Two. All we can point him/her/them to are the plateaus the students’ progress through in the story. They hint that the University may level people up only by mirroring and legitimating the tiered natures of its pre and post realms. To the wider point, we acknowledge that a problem of diversity statements or sections remains in that they do not solve problems, they only list types of things without much explanation, and even at that, as we have tried to show, the types of things are not always what we think. As we read texts, we project our own problems into them in search of answers to them, problems of who we are or who we want to help. Hopefully, this story was useful to you to that end.

## Conclusion

At the outset, we asked what books are. We looked at this through the methodological device of storytelling. We touched on some strange data that imply that books and stories may be agentic. We asked whether books are like humans. Our contribution was to attempt to show entanglement. Of how our humanity is not what we think it to be. It seeps into us from other things and backs out of us into other things: such as books, bells and blackbirds. The research study at the heart of the story hinted at why books may become so dangerous in the future—at what they might give us—the promise of some kind of intimacy. Perhaps the ultimate intimacy that we all need: a space for a willingness to be open and vulnerable to ourselves for a while.

If books are our emotional companions, then knowledge is not what we think it is. To say that knowledge is locked up in books or behind paywalls is to miss the point. It is situated. It sits, on the tarmac, reading us. Derrida (1982) would only grant his autumnal swirl of text sheaves but here we fitted them with spines and into books. The scholar, in interpreting the student testimony of the codex, tries to create a world for them. He tries to imagine books and petrol stations. Although we may feel sympathy for him in his bookless future, he creates a loving depiction of our world. We may feel anything from discomfort to shame in the present about our addictions to petrol and white male book knowledge, but to the scholar, we live in a utopia. It is never too late to re-imagine our world it seems.

It is getting late here, however. At the outset, we said that stories should not need to introduce themselves. We echoed Spivak (1998), in her anguish that a text should need a preface to ‘abstract so-called themes’ and rob a text ‘of its self-moving structure’ (while, of course, she was writing a preface). Similarly, as we fret for something to conclude upon, we will slip beyond text, in the manner of a footnote, to reassure you that ‘books are not like people’.^1^

## Footnotes

1 See John (2021): Books are not like people. https://open.spotify.com/album/6VXU4H145HtDNLJRZSRsk6. Accessed 18 June 2021.
